# Treatment of Milk-Sickness

**Published:** 1841-09

**Authors:** 


### TREATMENT OF MILK-SICKNESS.

Dr. John Evans of Attica, Indiana, has sent us a short account of a method of treating this disease, pursued for some years past by Dr. Wilson of that town, and lately by himself, which he affirms is almost invariably successful. The prescription is as follows:

℞ Pulv. Rhei ℥i.
Magnes. Cal. ℥ss.—Mix.

A table spoonful to be given in mucilage every two hours, till purging is produced. If vomited up, a new dose must be immediately administered. D.

